# Adapting the ADS Plus Program and Study for a Spanish Speaking Population

**DOI:** 10.1093/geroni/igab046.1870

**Published:** 2021-12-17

**Authors:** Elma Johnson, Katherine Marx, Manka Nkimbeng, Joseph Gaugler, Laura Gitlin, Lauren Parker

**Affiliations:** 1 University of Minnesota School of Public Health, University of Minnesota/ Minneapolis, Minnesota, United States; 2 Johns Hopkins School of Nursing, Baltimore, Maryland, United States; 3 University of Minnesota, Minneapolis, Minnesota, United States; 4 Drexel University, College of Nursing and Health Professions, Drexel University, Pennsylvania, United States; 5 Johns Hopkins Bloomberg School of Public Health, Baltimore, Maryland, United States

## Abstract

While Hispanic/Latinos are at increased risk for Alzheimer’s Disease, they are often cited as a “difficult-to-reach population” to engage in community-based research or clinical trials. One reason may be that many community-based supportive interventions for dementia caregivers are not adapted for Spanish-speaking populations. The purpose of this presentation is to describe the process of adapting the Adult Day Services Plus (ADS Plus) program for this population. In addition to translating ADS Plus into Spanish, staff, familiar with the program from four sites, which serve a predominantly Hispanic population, participated in a set of three focus groups that reviewed recruitment and intervention materials. Emerging themes included, Hispanic caregivers do not refer to themselves as caregivers but as the familial relationship (e.g. daughter, son, wife), and Hispanics often view research as a waste of resources. Future studies should consider these cultural elements towards caregiving in developing programs for Spanish-speaking dementia caregivers.

